# Supplementary material for: Challenges faced by human resources for health in Morocco: A scoping review
Source: PLoS One. 2024 May 7;19(5):e0296598. doi: 10.1371/journal.pone.0296598 (PMC11075827; doi:10.1371/journal.pone.0296598)
Supplement: S1 Appendix — (DOCX) [file pone.0296598.s001.docx]

**S1 Appendix (Search strategy applied to databases)**

| **Concepts** | Human resources for health; challenges; Morocco |
| --- | --- |
| **Synonyms/other keywords** | Human resources for health; health professionals; health providers; health workers; health personnel; health care delivery; nurses; doctors.  Challenges; barriers; shortage; opportunities; satisfaction; stress; burnout; occupational health.  Morocco; Maroc. |
| **Truncation** | challeng*; Morocc* |
| **Year** | 2014 up to 2023 |
| **Databases** | 1. PubMed 2. ScienceDirect 3. Google Scholar |
| **Studies to be included** | No limitation for type of study |
| **Inclusion criteria** | Articles addressing the challenges faced by human resources for health in any geographical region in Morocco will be included in this study.  Publications written in English or French languages will be eligible.  The context of all included studies will be Morocco. |
| **Exclusion criteria** | Studies with participants did not refer to human resources for health or if did not address a challenge context will be excluded. Also, any participant/study from a country other than Morocco will be excluded.  Publications will be excluded if they are relevant to the COVID-19 pandemic. |
