# Supplementary material for: Challenges faced by human resources for health in Morocco: A scoping review
Source: PLoS One. 2024 May 7;19(5):e0296598. doi: 10.1371/journal.pone.0296598 (PMC11075827; doi:10.1371/journal.pone.0296598)
Supplement: S3 Appendix — (DOCX) [file pone.0296598.s003.docx]

**S3 Appendix (Data extraction sheet)**

| **Category** | **Type of data** |
| --- | --- |
| 1. Bibliographic information | a. Author  b. Year of publication  c. Title of study  d. Aims of study  e. Region of study (setting)  d. Source/journal. |
| 2. Study design | Type of study, type of document, methodology used, and study population. |
| 3. HRH challenge | Type of HRH challenges. |
| 4. key finding | Main outcomes and results relevant to the research question. |
| 5. Proposed solution | Main recommendations and/or solutions proposed by authors to deal with HRH challenges. |
