# Supplementary material for: Challenges faced by human resources for health in Morocco: A scoping review
Source: PLoS One. 2024 May 7;19(5):e0296598. doi: 10.1371/journal.pone.0296598 (PMC11075827; doi:10.1371/journal.pone.0296598)
Supplement: Attachment — Submitted filename: Responses to the reviewers.docx [file pone.0296598.s005.docx]

**Responses to reviewers**

Dear Editor,

Thank you for giving us the opportunity to submit a revised draft of the manuscript for publication in the PLOS One journal. We appreciate the time and effort that you and the reviewers dedicated to providing feedback on our manuscript and are grateful for the insightful comments on and valuable improvements to our paper. We have incorporated most of the suggestions made by the reviewers. Those changes are highlighted within the manuscript. We hope the manuscript after careful revisions meet your high standards.

Please see below, in blue, for a point-by-point response to the reviewers’ comments and concerns.

Sincerely,

**Reviewer #1:**

[**General** **Comment**] This is a well written manuscript. It highlights the challenges faced by human resources for health in the context of Morocco. This manuscript highlights the lack of policies related to the HRH. This gives concise information about the problem among HRH on a personal level as well as institutional level. This will be helpful for concerned authorities while making new policies for health professionals. This manuscript also compares those problems with other developing nations as well as the developed nation.

**Response**: Thank you very much.

[**Comments embedded in the Manuscript**]

[**Comment 1**] P2L27: Should study consisting any personal other than doctor and nurses be included under multiple HRH.

**Response:** Thank you for your insightful comment regarding the inclusion of health professionals other than doctors and nurses in our study. We acknowledge the importance of recognizing the diverse roles within the Human Resources for Health (HRH) beyond the traditional categorization of solely doctors and nurses. Our intention was to encompass a broad perspective on HRH to reflect the multifaceted nature of healthcare provision, which indeed relies on a wide range of professionals, including but not limited to, allied health workers, technicians, midwives, pharmacists, and support staff.

[**Comment 2**] P3L56: HRH only.

**Response:** Revised accordingly.

[**Comment 3**] P6L98: Fig.1.

**Response:** Revised accordingly.

[**Comment 4**] P6L102: What are those three considerations?

**Response:** Thank you for bringing this matter to our attention. The three considerations are:

Q1- Is the HRH challenge described?

Q2- Are the research objectives clearly outlined?

Q3- Are the proposed solutions feasible?

[**Comment 5**] P7L117: The result says that the challenge surged between 2017 to 2023. How did the author made sure that the surge between 2019-2023 was not because of COVID outbreak.

**Response:** Thank you for your astute observation regarding the surge in challenges identified between 2017 and 2023, and the potential impact of the COVID-19 outbreak on these findings. We recognize the importance of distinguishing between the underlying trends in HRH challenges and the exacerbation of these challenges due to the pandemic. To ensure that our analysis accurately reflects the surge in challenges independent of COVID-19, we implemented the following measures:

1. **Temporal Analysis:** We conducted a temporal analysis of the challenges identified in the literature before and after the onset of the COVID-19 pandemic. This allowed us to establish baseline trends in HRH challenges prior to 2019 and assess whether the surge observed from 2019 to 2023 significantly deviated from these trends.
2. **Thematic Categorization:** We categorized the challenges identified in the literature into themes related to both pandemic-specific and non-pandemic factors. This enabled us to isolate challenges that were likely to have been exacerbated by the pandemic from those that represented ongoing issues within the HRH sector.
3. **Expert Consultation:** We consulted with experts in healthcare and public health to gain insights into the multifaceted nature of HRH challenges during the specified period. Their expertise helped us discern whether the identified surge could be attributed solely to the pandemic or to a broader array of factors.
4. **Cross-Referencing with Pre-Pandemic Data:** By cross-referencing our findings with data and reports published before the pandemic, we were able to identify consistent patterns and challenges that predated COVID-19. This helped confirm that the surge in challenges was not solely a result of the pandemic.

Through these measures, we aimed to provide a nuanced analysis that acknowledges the impact of COVID-19 while also recognizing the broader trends in HRH challenges. We believe this approach strengthens the validity of our findings and contributes to a comprehensive understanding of the HRH landscape from 2017 to 2023.

[**Comment 6**] P7L121: You mentioned 13 regional and 4=2=6 as national level. This altogether makes 19. The very next line is that 4 studies did not mentioned region of study. Are these 4 among 19 or others? If other are they in Fig 1 and Table 1 or 2.

**Response:** Thanks for your kind reminders. To remove this ambiguity, we replace it with the new one: “*Out of the studies reviewed, four publications did not specify their geographical focus. In contrast, eleven studies clearly identified the regional scope of their investigation, and the remaining four explored challenges at the national level.*”

[**Comment 7**] P7L121: How many out of 19?

**Response:** Revised accordingly (14/19).

[**Comment 8**] P7L123: Good to mention one of the study was focused exclusively for doctors.

**Response:** Revised accordingly.

[**Comments 9**] Table 1.

**Response:** Thank you very much for your nice reminder. We considered what is the appropriate presentation for this table. Finally, we used a separate column to present the sample size.

As suggested, nurse and assistant are replaced by single word “paramedics”.

We made a uniformity between "and" and "&", as requested.

[**Comment 10**] P9L129: Type 2 Diabetes (T2D).

**Response:** Revised accordingly.

[**Comment 11**] P9L136: Citations.

**Response:** All necessary citations have been incorporated into the text as requested.

[**Comment 12**] Table 2: You can add increase manpower as one of the proposed solutions.

**Response:** Revised accordingly.

[**Comment 13**] Table 2: General practitioners (GPs).

**Response:** Revised accordingly.

**[Comment 14**] Table 2: relationship with the immediate supervisor, relationship with colleagues, administrative support, level of responsibility, and working hours.

**Response:** Thank you very much for this suggestion which, been incorporated into the text as requested.

[**Comment 15**] Table 2: cannot find it on-line to verify.

**Response:** Thank you for your observation regarding the difficulty in verifying the document online. I understand the importance of accessing sources for validation purposes. The document in question is not yet published and, as such, is not available through online platforms or databases. This document was obtained through personal communication (under preparation for publication), and has been cited in our manuscript based on its relevance and the valuable insights it offers to our study.

To assist in the review process and ensure the integrity of our references, I am prepared to provide a copy of the document for review purposes if permissible by the document's authors. Alternatively, I can provide a detailed summary of the relevant sections that support our arguments and findings in the manuscript. Please advise on how you would prefer to proceed, and I will do my utmost to accommodate your request, respecting the confidentiality and distribution rights associated with the document.

[**Comment 16**] Table 2: of what?

**Response:** It is for personnel shortage. It has been incorporated into the text.

[**Comment 17**] P14L165: Good (30).

**Response:** Thank you for your comment. We have meticulously reviewed the studies referenced as (31) and (32) and concluded that both address the topic of 'dissatisfaction,' albeit from slightly different perspectives. Specifically, study (31) delves into the organization of health services at health centre levels, which we recognize as a crucial aspect of healthcare professionals' roles that can influence their satisfaction. This examination aligns with our broader investigation into factors contributing to dissatisfaction within the healthcare sector. Thus, we believe that including these studies enriches our analysis by showcasing the multifaceted nature of dissatisfaction among healthcare professionals.

[**Comment 18**] P16L217: Reference #47 is based in USA which is first world country. Do you think this is relevant for Morocco.

**Response:** Thank you for your insightful query regarding the relevance of reference 47, which is based in the USA, to the Moroccan context. We acknowledge the significant differences between healthcare systems, economic statuses, and societal structures of the USA and Morocco. However, we believe the inclusion of this reference offers valuable insights for several reasons:

1. **Universal Themes:** The core issues addressed in the study, such as training practices in medical education, are not unique to high-income countries. These themes have global relevance, including for middle- and low-income countries like Morocco, as they highlight universal challenges and potential solutions in healthcare workforce optimization.
2. **Best Practices and Innovations:** The study presents innovative approaches or best practices in Quality improvement education. By examining these, we can consider their adaptability and potential for implementation in Morocco, taking into account the necessary adjustments to fit the local context.
3. **Comparative Analysis:** Including studies from diverse economic backgrounds enriches our understanding and facilitates a comparative analysis. It encourages the exploration of how different healthcare systems tackle similar issues, providing a broader perspective that can inspire context-specific adaptations in Morocco.
4. **Evidence-based Decision Making:** Our goal is to base our recommendations on a wide range of evidence, drawing from various contexts to propose the most comprehensive and adaptable solutions for the Moroccan healthcare system.

[**Comment 19**] P16L217: Citation 4 consider disability as one of the problem where HRH might need to offer home to home facility but citation 52 and 53 did not talk anything about disability.

**Response:** Thank you for your attentive reading and valuable feedback regarding the cited references. We appreciate your observation that the first citation specifically addresses the issue of disability and its implications for HRH, including the potential need for home-based healthcare services. This reference was indeed selected to highlight the evolving challenges within health systems, including the increasing need to address disability more comprehensively.

Regarding the subsequent citations from Frenk et al. (2010) and Tsiachristas et al. (2015), you are correct in noting that these works do not explicitly discuss disability. These references were included to provide a broader perspective on the challenges and changes impacting healthcare systems, HRH roles, and educational policies globally. While they do not specifically address disability, they contribute to the overarching narrative of our study by illustrating the need for healthcare systems, including Morocco's, to adapt to a wide range of evolving demands; of which disability care is an important part.

Our intention in citing these works together was to underscore the complex and multifaceted nature of the challenges facing HRH in the current era, which includes but is not limited to, the specific issue of disability. The broader aim is to advocate for a comprehensive approach to HRH training and policy reform that can accommodate diverse healthcare needs, including the nuanced care requirements of people with disabilities.

We hope this clarifies our rationale for including these citations and how they collectively inform the discussion on HRH challenges and potential solutions in the face of significant healthcare transitions.

[**Comment 20**] References 3, 5, 29, 45, 54 and 64: Do you need cited 2023 Sept 23?

**Response:** Thank you for your suggestion to eliminate this information.

**Reviewer #2:**

[**Comment**] It followed rigorous methodology that has yielded into good study findings and conclusion. Though, study data is available, the data extraction sheet for selected papers could also be provided. Authors can also elaborate about the consultations done to develop the research question.

**Response** *“It followed……conclusion”*: Thank you very much.

**Response** *“Though, ….. be provided.”*: Thank you for your suggestion to provide the data extraction sheet for the selected papers. We appreciate the importance of transparency and detail in presenting our methodology to facilitate understanding and replication of our study.

In line with your recommendation, we have prepared a supplementary file that includes the data extraction sheet utilized during our review process. This sheet comprehensively outlines the criteria and variables used to collect and analyse data from the selected papers, ensuring a clear understanding of our methodological approach.

The data extraction sheet includes the following key elements:

1. **Bibliographic information:** Author(s), year of publication, title, aims, setting and source/journal.
2. **Study Design:** Type of study, type of document, methodology used, and study population.
3. **HRH challenge:** Type of HRH challenges.
4. **Key Findings:** Major outcomes and results relevant to the research question.
5. **Proposed solution:** Main recommendations and/or solutions proposed by authors to deal with HRH challenges.

The supplementary file is attached for your review and has been referenced in the revised manuscript for the convenience of future readers (S3 appendix).

**Response** *“Authors can ….. question.”*: Thank you for your insightful query regarding the consultations undertaken to develop our research question. In the initial phase of our study, we recognized the importance of grounding our research question in a robust understanding of the field and the specific needs of the population we aimed to study. To this end, we engaged in a multi-step consultation process:

1. **Expert Panel Consultation:** We assembled a panel comprising seasoned professionals from the health ministry, representatives from the Eastern Mediterranean Regional Office (EMRO), and academicians with extensive experience in health research. This panel provided critical insights into the current challenges and gaps in the health sector, guiding the formulation of a relevant and impactful research question.
2. **Literature Review:** A comprehensive review of existing literature was conducted to ensure our research question addressed an unmet need in the field. This step helped refine our understanding and provided a solid foundation for our study's focus.
3. **Stakeholder Meetings:** Meetings with healthcare providers, policy-makers, and potential beneficiaries of our research were held to gather diverse perspectives on the pressing issues in the health sector. These discussions were invaluable in identifying areas where further inquiry was needed and ensuring our research would be practical and applicable.
4. **Feedback Loop:** The draft research question was circulated among the expert panel and stakeholders for feedback. This iterative process allowed us to refine and adjust our question based on expert critique and suggestions, ensuring it was both scientifically rigorous and aligned with the needs of the healthcare community.

This thorough and inclusive approach to developing our research question has ensured that our study addresses a critical gap in the field and is poised to contribute meaningful insights into optimizing the health workforce to meet the evolving healthcare needs of the Moroccan population.

**Reviewer #3:**

[**Comment**] The effort committed into the manuscript is appreciated. However, authors may wish to note that the manuscript has not clearly answered the research question. In addition, the flow of the manuscript needs to be carefully worked on to appeal to readers. In the table, separate column should be used, each for type of study and the sample size. It seems the manuscript has been published on a blog (accessed -30-1-24) <https://sciety.org/articles/activity/10.1101/2023.12.21.23300411>

**Response** *“The effort……appreciated”*: Thank you very much.

**Response** *“However ……* *to readers”*: Thanks for your kind reminders. We appreciate your observation and understand the concern regarding the clarity with which our research question has been addressed in the manuscript. In response, we would like to clarify that the methodological nature of a scoping review inherently guided our approach to analysing and presenting the findings. As scoping reviews aim to map the extent, range, and nature of research activity in a broad field, our study necessarily adopted a narrative approach to synthesize and interpret the extensive body of literature we reviewed.

This narrative synthesis allowed us to explore and present a comprehensive overview of the challenges and potential solutions within the health workforce optimization field, aligning with the broad and exploratory nature of our research question. While this approach facilitated a wide-ranging exploration of the topic, we acknowledge that it may have limited our ability to provide a pointed answer to the research question that resembles the specificity often found in systematic reviews or meta-analyses.

**Response** *“In the table, ….. sample size.”*: Thanks for your kind reminders. We revised the table accordingly.

**Response** *“It seems …. blog”*: Thanks for your comment. To clarify, the content that appeared on the mentioned blog represents a pre-publication dissemination of our findings. This action was taken in parallel with our submission to your journal and was guided by a recommendation found within the journal's guidelines for authors. The intent behind this parallel dissemination was to engage with the wider scientific community early on, fostering an open exchange of ideas and feedback on our preliminary findings.

We understand the importance of maintaining the integrity of the publication process and assure you that the blog post was intended as an additional avenue for scientific dialogue, not as a substitute for formal peer-reviewed publication. We have strictly adhered to the journal's guidelines in this process, ensuring that our actions promote transparency and constructive scientific discussion without compromising the originality and review process of our submission.
